# Dataset on a health education promoting breast self-examination among women in Indonesia

**DOI:** 10.3389/fpubh.2025.1569763

**Published:** 2025-05-20

**Authors:** Triana Kesuma Dewi, Patrycja Figarska, Teresa Lutz, Muchlisah Audina Sudirman, Nabila Nasywa Rasyiidah, Melisa Nurcahya Tappi, Robert A. C. Ruiter

**Affiliations:** ^1^Clinical and Health Psychology Research Group, Faculty of Psychology, Universitas Airlangga, Surabaya, Indonesia; ^2^Department of Work and Social Psychology, Faculty of Psychology and Neuroscience, Maastricht University, Maastricht, Netherlands

**Keywords:** breast cancer, intervention mapping, social determinants of health, attitude, perceived barriers, subjective norms, self efficacy, knowledge

## 1 Background

Breast cancer remains a significant public health issue in Indonesia. In 2022, breast cancer became the most commonly diagnosed cancer among women, accounting for 30.1% of all cases, and ranked as the third leading cause of cancer-related deaths (1). Alarmingly, only 2% of breast cancer cases were diagnosed at an early stage, while ~70% of patients sought treatment only when the disease had progressed to advanced stages (stages III and IV). This late diagnose results in more challenging, costly, and complex treatment and worsens patient prognoses (2, 3). Therefore, a robust approach to achieving early detection and treatment is necessary to improve outcomes for this potentially curable disease (2).

Breast cancer early detection involves efforts to identify and diagnose the disease at an early stage (4). Breast self-examination (BSE) followed by prompt medical evaluation upon detecting abnormalities are considered as the practical alternatives to achieve breast cancer early detection in Low-and-Middle-Income Countries [LMICs; see (5, 6)]. BSE is a self-screening method that combines physical and visual breast examinations to detect changes or signs of breast cancer (7). Regular practice of BSE is linked to increased health-seeking behaviors and higher rates of undergoing biopsies, leading to earlier diagnoses and improved prognoses (8, 9). Despite its benefits, the prevalence of BSE practice among Indonesian women remains low (10, 11). As such, health interventions promoting BSE among Indonesian women need to be initiated and strengthened.

In response, we aim to develop a health education to promote BSE practices among women in Indonesia. Considering the Indonesian's unique cultural context, including traditional and religious values (12, 13), tailoring the intervention to Indonesian women is crucial for ensuring its feasibility and effectiveness. The Intervention Mapping (IM) protocol (19) serves as the framework for this initiative. IM is a structured, systematic, evidence-based, and participatory approach for designing, implementing, and evaluating behavioral change programs. The process comprises six iterative steps: 1) developing a logic model of the problem, 2) creating a logic model of change, 3) program design, 4) program production, 5) program implementation planning, and 6) evaluation planning. Previous studies have demonstrated the effectiveness of IM in developing intervention programs for health behaviors, including breast and cervical cancer screening (14, 15) and colorectal cancer screening (16). This article aims to present the dataset and intervention materials developed to promote BSE among Indonesian women.

## 2 Methods

The current study utilized a quasi-experimental design with three measurement phases: pre-test, post-test, and 1-month follow-up to assess the effectiveness of a health education focusing on promoting BSE among women, specifically in Surabaya, Indonesia. Ethical approval was obtained from the Faculty of Nursing Ethical Board, Universitas Airlangga. Participation in the study was anonymous and voluntary, and all of the participants provided their written informed consent before participation. Initially, there were 89 participants consented to participate in the study, however, a final of 70 participants joined the study up to the follow-up phase (21.35% dropout rate), which was later included in the final analysis.

The study used a combination of purposive and snowball techniques to collect the data: the inclusion criteria were: 1) Women aged 18–55 years, 2) Lived in Surabaya for at least 1 year, and 3) No history of breast cancer. Women with an experience of attending a similar training in the past year were excluded from the study. The invitation to join in the experimental study was shared on the university, PKK^1^, and research team's social media (e.g., Instagram, Facebook, WhatsApp, and Twitter) which was further forwarded by colleagues and some of the participants to their networks. At the end of the experimental study, each participant of both active and passive groups was provided with an e-wallet worth IDR 200.000 as a token of appreciation. Additionally, the participants of active groups (who attended the health education in person) were also provided with snacks, lunch, and an IDR 100.000 transportation fee.

The study was first designed as a three-arms experimental study, involving 3 conditions: 1) Active group—action planning, who will receive a face-to-face intervention focusing on active planning strategy, 2) Active group—implementation intention, who will receive face-to-face intervention focusing on implementation intention strategy, and 3) Passive group who will receive self-study digital booklet. Before the data collection, the sample size estimation was calculated by using G^*^Power (17) estimating 24 participants per condition and 72 participants in total. The estimation of the sample size was based on power analysis with a power of 0.80 for three groups, an expected effect size of 0.43 derived from a similar study assessing the effectivity of self-help intervention for breast cancer prevention in Indonesia (18), and an estimated drop-out rate of 25%.

Once a participant consented to participate in the study, she will be directed to information that they can be chosen to attend a face-to-face health intervention on the stated date and time in our university. If they agree, they may proceed with filling out the pre-test. Later, the participants who filled out the pre-test were randomly assigned into three study groups using software: https://www.random.org/sequences/ to ensure simple randomization. Once the randomization results were generated, the researcher contacted the participants via their registered phone or WhatsApp number to inform their assignment and specifically, to ensure their availability to attend the face-to-face health intervention for those who were assigned in the active groups. Due to further participants' confirmation (i.e., several active group participants confirmed that they could not attend the face-to-face health intervention), we offered them to join the passive group, and consequently, we also offered the same number of participants in the passive group to join in the active groups. Thus, resulting in an adjustment of the confirmed groups' members. However, on the day of the health education, the number of participants who showed up was not sufficient to form two active groups. Therefore, we decided to merge the active groups by delivering a health intervention focusing on action planning only (further, this group was referred to as an active group). Therefore, the final groups were formed based on the pragmatic factors.

The active condition group attended a 3-h face-to-face health intervention at the Faculty of Psychology, Universitas Airlangga. The health intervention was facilitated by a trained female instructor in a private-classroom setting, to ensure their comfortability to perform BSE. In the first 30 min, breast cancer knowledge and breast cancer literacy were explained and discussed with the participants, supplemented with an activity to assess participants' personal risk factors for breast cancer. In the next 15 min, participants were presented and asked to reflect on breast cancer survivor's perspectives and experiences related to breast cancer early presentation including BSE. A 20-min session was dedicated to practicing BSE guided by a video of BSE step-by-step strengthened with feedback from the instructor. Further, participants were asked to reflect on how would their life be with and without BSE and discussed it in the classroom. Finally, making a detailed plan for performing BSE at home for the next 3 months was practiced by the participants. At the end of the health intervention, participants were asked to fill out the post-test and share a digital booklet containing a detailed description of the health intervention to read at home. Further details of the instructor guideline can be found at https://osf.io/wcefb/files/osfstorage, under “5. Program Implementation Plan.”

The passive condition group received a digital version of the workshop booklet similar to those received by the active groups, shared via their registered WhatsApp number. They were given 4 days to finish the booklet and finally asked to fill out the post-measurement which link was attached to the end page of the booklet.

## 3 Data description

The dataset provides information on the evaluation of the effectiveness of a health education focusing on promoting BSE among women in Indonesia. The pre-registration of the experimental study can be found at https://osf.io/mxh5r. The health education materials, questionnaires, and raw data are publicly available through OSF under the project titled “*The Development of Breast self-examination health Intervention among women in Indonesia: An Intervention Mapping Approach*”. We developed the education materials, including a presentation of the workshop, worksheet, and booklet for the participants consisting of theoretical and practical information about the activities presented in the health intervention program which is developed by using the Intervention Mapping approach (19). A full overview of intervention materials can be found at https://osf.io/wcefb/files/osfstorage, under the title “*4. Program Production*.”

The online questionnaires used to collect the data were adapted from Dewi (20) which included the measurement of intention to perform BSE, attitudes toward breast cancer and BSE, subjective norms, perceived behavioral control, and perceived barriers toward performing BSE. All measures used a 5-point Likert scale. Additionally, breast cancer knowledge and BSE literacy (answer options Yes, No, or I don't know) were also assessed. Finally, the frequency of BSE practice, as well as sociodemographic variables (i.e., age, marital status, residential area, occupation, educational level, monthly income, family history of breast cancer, and participation in previous BSE intervention) were assessed. The data measurements were conducted during the pre-test, post-test, and 1-month follow-up. However, the pre-test measurement used the short version of the post-test/follow-up questionnaire to minimize the *carryover effects* [i.e., the possibility of pre-test measurement alters the response in post-test measurement in a repeated measurement design; see Neuman (21)]. Table 1 provides the overview of the scale (i.e., the pre-test and post-test/follow-up); further details of the questionnaire can be accessed at https://osf.io/wcefb/files/osfstorage under the title “*6. Evaluation Plan*.”

**Table 1:** The summary of scales.

**Scales**	**Number of items**		**Alpha Cronbach's**		**Example of the item**
	**Pre-test**	**Post-test**	**Pre-test**	**Post-test**	
Intention	3	3	0.70	0.53	I intend to perform BSE monthly as recommended.
Attitudes	8	8	0.59	0.77	I believe that performing BSE monthly is an important thing to do.
Perceived barriers	3	3	0.40	0.63	I am confident that I could perform monthly, even if I have other competing priorities (e.g., childcare, employment, family commitments,...).
Subjective norms	6	6	0.73	0.8	I believe that most people who are important to me approve of my performing BSE monthly.
Perceived behavioral control	5	5	0.81	0.85	I believe I could perform BSE monthly with the correct procedures.

The study was a two arms experimental study carried out between May and June 2024. A final of 70 participants joined in the study, distributed to the active group (*n* = 36) and passive group (*n* = 34). The sociodemographic information of the participants is presented in Table 2.

**Table 2:** Socio-demographic characteristics of the participants.

**Socio-demographic characteristics**	**Overall**		**Active**		**Passive**		**Statistics**
	**n**	**n/N%**	**n**	**n/N%**	**n**	**n/N%**	
**Marital status**							*X^2^* (1) = 0.02, *p* = 0.886
Yes	17	24.55	9	12.86	8	11.43	
No	53	75.7	27	38.57	26	37.14	
**Family history of breast cancer**							*X^2^* (2) = 1.02, *p* = 0.6
Yes	6	8.6	4	5.71	2	42.86	
No	52	74.3	25	35.71	27	38.57	
I don't know	12	17.1	7	10	5	7.14	
**Health insurance**							*X^2^* (1) = 0.02, *p* = 0.886
Yes	53	75.7	27	38.57	26	37.14	
No	17	24.3	9	12.86	8	11.43	
**Occupation**							*X^2^* (5) = 78.11, *p* = 0.15
Student	37	52.9	21	30	16	22.86	
Unemployed	7	10	5	7.14	2	2.86	
Private sector employee	11	15.7	5	7.14	6	8.57	
Civil servant	2	2.8	0	0	2	2.86	
Informal worker	4	5.7	0	0	4	5.71	
Other	9	12.9	5	7.14	4	5.71	
**Income** ^a^							*X^2^* (2) = 4.74, *p* = 0.093
Below standard	26	37.1	17	24.29	9	12.86	
Standard income	32	45.7	12	17.14	20	28.57	
High income	12	17.1	7	10	5	7.14	
**Education**							*X^2^* (3) = 8.24, *p* = 0.041^*^
Undergraduate	32	45.7	16	22.86	16	22.86	
Senior high school	28	40	17	24.29	11	17.51	
1–3 years diploma	4	5.7	3	4.29	1	1.43	
Postgraduate	6	8.6	0	0	6	8.57	
**BSE in last year**							*X^2^* (2) = 0.562, *p* = 0.755
Never	56	80	30	42.86	26	37.14	
Yes, no routine	12	17.1	5	7.14	7	10	
Yes, every month	2	2.9	1	1.43	1	1.43	

^a^Indonesia basic monthly income national standard is IDR 4,200.000. ^*^p < 0.05, *N* = 70.

## 4 Discussion

The presented dataset marks an initial publication on the effectiveness of a health education program to promote BSE among women in Indonesia. Specifically, the dataset provides information on sociodemographic as well as primary outcomes (i.e., BSE intention and behavior) and secondary outcomes (i.e., breast cancer knowledge; BSE literacy; perceived barriers, perceived behavioral control, subjective norms, and attitudes toward BSE). Therefore, further statistical analysis results will be beneficial for researchers, practitioners, or policymakers who propose to design a health education and/or an experimental study in this context. Additionally, the dataset may be use for comparison with a different population. Furthermore, the scales and information related to psychometric properties (i.e., Cronbach's Alpha) of the scales measuring intention, attitude, subjective norms, perceived behavioral control, and perceived barriers toward performing breast self-examination were also reported. Thus, it would be beneficial for practitioners or policymakers who aim to promote BSE practice as a first important step to achieve breast cancer early presentation.

## 5 Limitations

The study initially aimed to assess the effectiveness of a health education to promote BSE by comparing three strategies: focusing on action planning (active group—action planning), implementation intention (active group—implementation intention), and self-study (passive group). However, due to the adjustment of research methods from three to two arms experimental methods, the data cannot provide information on the effectiveness of the *implementation intention* strategy to promote BSE. Although the researcher provided the information about the intervention schedule (i.e., day, date, and time) before participation consent followed by reminder messages via WhatsApp to all of the participants, the drop-out rate was still high. This leads to the elimination of *active group–implementation intention*. Therefore, an advanced strategy to avoid participants dropping out in the future similar research should be explored. Additionally, due to the nature of how the research invitation was being disseminated, the participants were mostly coming from a university background. Therefore, covering specific socio-demographic characteristics: single, having graduated from high school, and being a university student. Although the sample size met power analysis requirements, the relatively small sample may limit external validity. Future studies involving larger and more diverse sample are recommended to improve generalizability. Additionally, the 1-month follow-up limits insight into long term behavior maintenance. Future study should incorporate longer follow ups periods to evaluate the sustainability of the intervention's effects over time.

## Data Availability

The datasets presented in this study can be found in online repositories. The names of the repository/repositories and accession number(s) can be found at: Repository name: Open Science Framework (OSF). Data identification number: DOI 10.17605/OSF.IO/WCEFB. Direct link to the dataset: https://osf.io/wcefb/.

## Footnotes

^1^PKK is a *Women's Family Welfare Movement', a nation-wide women's NGO that seeks to reach every wife in a certain geographic area*.
